# Stress Dynamics in a Basketball Game: Examining Stressors, Game-Specific Factors, and Players’ Positions

**DOI:** 10.3390/jfmk11030293

**Published:** 2026-07-26

**Authors:** Ivan Perasović, Antonela Karmen Ivišić, Dario Vrdoljak, Nikola Foretić, Vladimir Pavlinović, Ratko Perić, Mia Perić, Zoran Nikolovski

**Affiliations:** 1 Faculty of Kinesiology, University of Split, 21000 Split, Croatia; ivan.peraso@gmail.com (I.P.); nela.ivisic97@gmail.com (A.K.I.); nikola.foretic@kifst.eu (N.F.); vladimir.pavlinovic@kifst.eu (V.P.); mia.peric@kifst.eu (M.P.); 2 School of Health & Exercise Sciences, University of British Columbia, Okanagan Campus, Kelowna, BC V1V 1V7, Canada; dariovrdoljak42@gmail.com; 3 Centre for Heart, Lung and Vascular Health, University of British Columbia, Okanagan Campus, Kelowna, BC V1V 1V7, Canada; 4 Department of Exercise Physiology, Orthopaedic Clinic Orthosport, 78000 Banja Luka, Bosnia and Herzegovina; ratkoperic@yahoo.com; 5 High Performance Sport Center, Croatian Olympic Committee, 10000 Zagreb, Croatia

**Keywords:** salivary cortisol, salivary alpha-amylase, HPA axis, SAM system, internal load, player position, professional basketball

## Abstract

**Background**: This study investigates the psychophysiological stress profiles of professional basketball players by simultaneously analyzing two distinct neuroendocrine pathways: the sympathetic–adrenal–medullary (SAM) system, via salivary alpha-amylase (AA), and the hypothalamic–pituitary–adrenal (HPA) axis, via salivary cortisol (C). While competitive environments are known to elicit significant stress, the interaction between game-specific variables—such as playing time (PT) and tactical position—and those biomarkers remains insufficiently explored in real-time, official match settings. **Methods**: Twelve professional male basketball players were monitored across four official matches. Salivary samples were collected, and stress biomarkers (AA and C) were analyzed at three timepoints: pre-match, half-time, and post-match. **Results**: Results indicate that stress responses are non-uniform and highly context-dependent. A significant spike in AA was observed during the first match (*p* < 0.001; ES = 0.82; 95% CI −0.01–1.66), where concentrations rose from a pre-match mean of 247.9 ± 232.59 U/mL to a post-match peak of 674.39 ± 693.33 U/mL. Cortisol responses did not vary significantly across matches but exhibited a strong positive correlation with PT (*p* = 0.01; R = 0.76), identifying that C may serve as a primary marker of individual physical workload. Positional analysis revealed significant divergence in HPA activation; guards (0.55 ± 0.31 µg/dL) and centers (0.51 ± 0.22 µg/dL) exhibited higher cortisol levels compared to forwards (0.24 ± 0.18 µg/dL), reflecting the high cognitive/decision-making load of guards and the mechanical/physical contact load of centers. **Conclusions**: The observed differences in AA and C liberation suggest that stress responses during matches are influenced by a complex interaction of physiological and psychological stimuli characteristic of competitive basketball. Consequently, these findings indicate the importance and value of a dual-biomarker approach which might provide a framework for optimized training load management and recovery protocols in elite basketball.

## 1. Introduction

Competitive sports represent a complex and dynamic stress environment in which athletes are exposed to multiple interacting physiological and psychological stressors. During competition, athletes experience multidimensional demands that induce integrated psychophysiological responses influenced by factors such as physical workload, cognitive demands, pre-competitive anxiety, and situational pressure [1,2]. These responses are particularly evident in team sports, where performance depends on the interaction between physical output and decision-making under pressure. Basketball is a team sport characterized as a high-intensity intermittent activity requiring repeated accelerations, decelerations, rapid changes of direction, and frequent physical contact, combined with continuous perceptual–cognitive processing [3]. In addition to these physiological demands, players are exposed to substantial psychological stressors, including match importance, uncertainty of outcome, coach expectations, and opponent quality [4,5]. Consequently, basketball matches represent a potent stress stimulus due to the combined and interacting effects of physical and psychological load [6]. Recent evidence further confirms that basketball competition induces significant psychophysiological stress, reflected by acute changes in hormonal and salivary biomarkers [7]. The stress response in athletes is primarily mediated by two neuroendocrine pathways: the hypothalamic–pituitary–adrenal (HPA) axis and the sympathetic–adrenal–medullary (SAM) system [8]. Activation of the HPA axis leads to the secretion of cortisol (C), a hormone associated with metabolic regulation and adaptation to prolonged physical and psychological stress [9]. In contrast, the SAM system is responsible for rapid responses to acute stressors, with salivary α-amylase (AA) serving as a reliable biomarker of sympathetic nervous system activation [10]. Importantly, these systems may respond independently depending on the type, duration, and intensity of the stressor [11]. Cortisol responses are generally associated with sustained physical load and energetic demands, whereas AA is more sensitive to acute psychological stressors such as anxiety and anticipation [9]. Recent literature has emphasized the importance of simultaneously assessing multiple biomarkers to better understand stress responses in sport. A systematic review by Kamarauskas and Conte [7] reported that basketball matches consistently induce increases in salivary C and AA, highlighting the combined physiological and psychological stress experienced during competition [7]. Similarly, an updated systematic review published in 2025 demonstrated that salivary cortisol reliably reflects acute match-induced stress in basketball players, while hormonal responses may vary depending on contextual factors such as match conditions and training interventions. The study by Crewther et al. [12] found that salivary cortisol responses to a standardized mid-week stress test differed according to subsequent match outcome, suggesting that winning and losing are associated with distinct physiological stress responses [12]. These findings indicate that match outcome (winning vs. losing) may influence athletes’ endocrine stress reactivity during international rugby union competition. These findings support the use of non-invasive salivary biomarkers as practical tools for monitoring athlete stress in real-world settings [13]. Although previous studies have consistently shown that competitive environments elicit significant hormonal responses, the magnitude and pattern of these responses appear to vary across studies. For instance, increases in cortisol following basketball matches have been observed regardless of match outcome, phase of competition, or location, indicating a generalized stress response to competition. However, variability in responses suggests that contextual factors such as opponent level, match importance, and individual player characteristics may influence biomarker dynamics [6,12]. Therefore, interpreting stress biomarkers in sport requires consideration of both situational and individual determinants.

Despite increasing research interest, a limited number of studies have simultaneously examined both cortisol and AA responses in real-game basketball settings. Most existing research has focused on isolated biomarkers or training environments rather than official matches [14,15]. Furthermore, there is a lack of studies investigating the influence of specific game-related variables on stress responses, such as playing time and playing position [16]. Playing time is a particularly relevant variable, as it reflects external load and cumulative exposure to match demands, which may influence both physiological and psychological stress responses [17]. Prolonged participation during matches is likely associated with increased metabolic strain and greater exposure to competitive stressors. In addition, basketball playing positions are characterized by distinct tactical roles, physical demands, and cognitive responsibilities. Guards are typically involved in play organization and decision-making, forwards balance offensive and defensive roles, and centers are exposed to greater physical contact and interior play [18]. These differences may contribute to position-specific stress responses, although this aspect remains insufficiently explored in the literature. Although previous basketball studies have examined salivary cortisol and, in some cases, alpha-amylase, these investigations have predominantly involved youth or adolescent players and/or training, simulated, or experimental match conditions, whereas evidence from professional senior players competing in official matches remains scarce. Therefore, the aim of the present study was to investigate changes in salivary stress biomarkers during professional basketball matches under competitive settings. Particular emphasis was placed on examining the responses of salivary C and AA, as well as their relationship with playing time, and potential differences across playing positions. A more comprehensive understanding of these relationships may contribute to the development of improved athlete monitoring strategies, enabling better management of training load, recovery, and stress, ultimately enhancing performance and reducing the risk of overtraining. We hypothesized that official basketball matches would elicit significant increases in salivary C and AA concentrations, that these responses would be positively associated with playing time, and that biomarker responses would differ according to playing position.

## 2. Materials and Methods

### 2.1. Participants

The participants in this study were 12 male basketball players (7 guards, 3 forwards, 2 center players; age 23.92 ± 4.19 years (all older than 18 years), height 195.53 ± 7.06 cm, body mass 90.53 ± 8.22 kg). All players were professional players on the professional basketball team of KK “Alkar” (Sinj) playing at the highest competitive level in Croatia, and they regularly trained for at least 20 h per week. The standard weekly training program consisted of 7 basketball sessions and 3 conditioning sessions. The study was conducted during one competitive season. Participation in this study was voluntary. All players were informed about the purpose of the study and signed an informed consent form prior to study, and procedures were approved by the Research Ethics Board (Ethics Board Approval No. 2181-205-02-05-23-024).

### 2.2. Procedures

In addition to anthropometric variables (body mass, body height), in this study, playing time and salivary biomarkers were analyzed. Body mass (measured in 0.1 kg) and body height (measured in cm) were measured using a stadiometer (Seca 213, Hamburg, Germany) and a digital scale (Seca 769, Hamburg, Germany). This study was based on measurements performed during four official basketball matches, consisting of four quarters of 10 min with a standard break of 10 min. Two matches were played at home and two away. The opposing teams were of comparable quality, as reflected by the average match score (84:78), the mean total points scored per game (159.4), and the fact that all four teams were ranked between 4th and 8th place in the league during the study period. The calculation of playing time is executed through a synchronized data entry protocol where on-site statisticians utilize mobile applications to record substitution timestamps in real-time. These precise entries and exit points are integrated into the Genius Sports ecosystem to generate standardized participation metrics for official league records. In this study, salivary cortisol and alpha-amylase concentrations were assessed before the match (pre-match; approximately 18:00), during the half-time break (half-time; approximately 19:45), and after the match (post-match; approximately 20:45). The sampling protocol was designed to align with the team’s regular training and competition schedule. Specifically, the final training session before each match was conducted on the fifth day of the training week (typically Friday at 18:00), followed by a rest day on the sixth day. The official match was then played on the seventh day (typically Sunday between 17:00 and 19:00), providing approximately 48 h of recovery between the final training session and the match. For the purpose of salivary marker determination, players were asked to avoid eating a major meal, not to consume caffeine, cola drinks, or supplementation 60 min before sample collection. Additionally, special attention was given to avoid strong brushing of teeth in order not to provoke bleeding of the gum, which might increase C level coming from the blood. Ten minutes before each sample was collected, athletes rinsed their mouth thoroughly with water. In this period, athletes were not allowed to consume any fluid in order to standardize the sampling procedure [11]. After the samples’ collection, participants were allowed to drink ad libitum. For sample collection, the SalivaBio Oral Swabs—SOS (Salimetrics LLC, State College, PA, USA) were used, placing them underneath the tongue on the floor of the mouth for 2 min. After collection, swabs were placed into a storage tube and were refrigerated immediately. Within 2 h following sampling, samples were frozen at below −20 °C until centrifugation. On the day of analysis, samples were thawed completely and centrifuged at 1500× *g* (3000 rpm) for 15 min. After centrifugation, assays were performed. Salivary cortisol was analyzed with a commercially available enzyme-linked immunosorbent assay (ELISA) purchased from Salimetrics LLC (State College, PA, USA) on a microplate reader (Infinite 200PRO, Tecan, Mannendorf, Switzerland). Standard curves were constructed according to the manufacturer’s instructions and commercially available standards, and quality control samples were used for all assays. The assay sensitivity for salivary cortisol was 0.007 µg/dL, with an average intra-assay CV of 4.5%. All samples were analyzed in the same batch to avoid interassay variability. Samples for alpha-amylase were analyzed using a kinetic enzyme assay kit (Salimetrics LLC, State College, PA, USA). The average intra-assay CV was 5.5%. Values are expressed as alpha-amylase concentration (U/mL). The measured concentrations were corrected for the salivary flow rate to minimize the influence of dehydration [11].

### 2.3. Data and Statistical Analyses

To monitor changes between matches, the timepoints (Equation (1)) and matches (Equation (2)) were included in area under the curve (AUC) calculations. These equations slightly differ from the traditional trapezoidal formula since the timepoints are equally distributed; precisely, all are Δx = 1, which allowed for a slightly modified equation. AUC was used as the best representative of a full match stress response for C and AA, since the biomarkers were collected at 3 timepoints as stated above. Also, to observe differences between playing position and relation to playing time, AUC was applied as the primary characterization.

(1) $$AUC=(\left(Pre-match\right)+2\times \left(Half\;time\right)+\left(Post-match\right))÷2$$

(2) $$AUC=(\left(1st\;match\right)+2\times \left(2nd\;match\right)+2\times \left(3rd\;match\right)+\left(4th\;match\right))÷2$$

Furthermore, AUC was calculated for every individual participant and subsequently used in the statistical analysis. The statistical analyses included descriptive statistics to calculate means and standard deviations. Additionally, ANOVA for repeated measurements was used to examine possible differences between timepoints and matches for both absolute and AUC values. This statistical method was chosen because it was previously determined to be the strongest for this analysis [19]. If a significant result was found, a further Bonferroni post-hoc test was calculated to establish the presence of significance among measured timepoints and matches. The same analysis was used to determine the existence of differences among playing positions. To fully assess the differences among measurements of salivary biomarkers, the magnitude-based Cohen’s effect size (ES) statistic with modified qualitative descriptors (trivial ES: < 0.2; small ES: 0.21–0.60; moderate ES: 0.61–1.20; large ES: 1.21–1.99; and very large ES: > 2.0 was used. Lastly, to understand the variables that mostly influence the fluctuation of biomarkers, we assessed the relationship between playing time and the AUC of all matches as well as the AUC of individual matches. This relationship was calculated by employing Pearson’s correlation, represented by the correlation coefficient (R), and a level of significance set at *p* < 0.05. Additionally, a post hoc power analysis was performed for the repeated-measures ANOVA. For AA, the main effect of Sample demonstrated a large effect size (ηp^2^ = 0.343) with excellent observed power (1 − β > 0.999). The main effect of Time showed a small-to-medium effect size (ηp^2^ = 0.057) with moderate observed power (1 − β ≈ 0.55–0.65), which is consistent with the non-significant result (*p* = 0.06). For C, the main effect of Sample demonstrated a large effect size (ηp^2^ = 0.154) with excellent observed power (1 − β > 0.99). The main effect of Time showed a medium effect size (ηp^2^ = 0.125) with high observed power (1 − β ≈ 0.95–0.98). All statistical and data analyses were conducted using R version 4.1.2 (Vienna, Austria), and figures were created using GraphPad software, v 10.

## 3. Results

**Figure 1:**
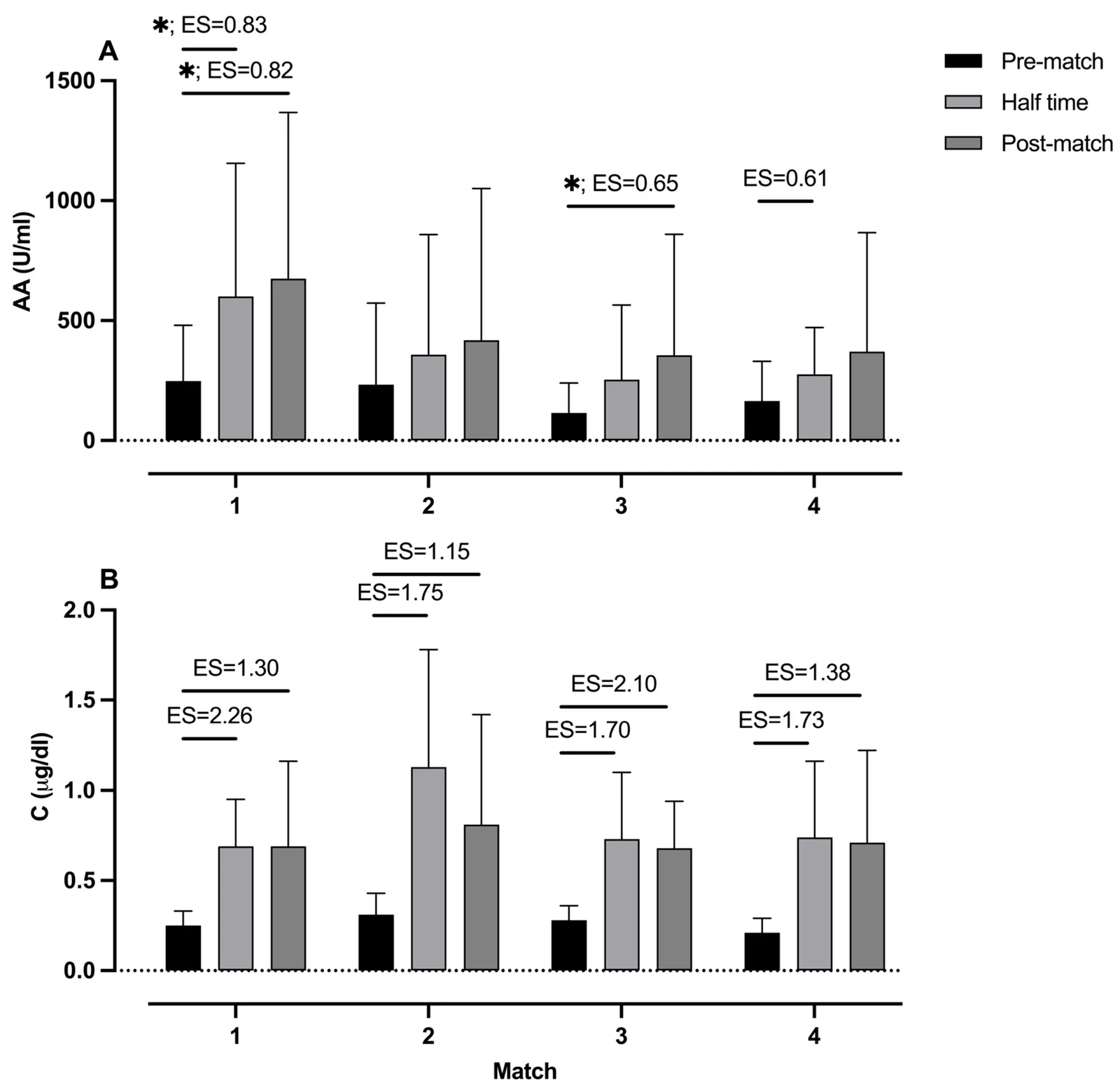
Descriptive statistics for concentrations of alpha-amylase (U/mL) (**A**) and cortisol (µg/dL) at all 4 matches (1 to 4) (**B**) pre-match, at the half-time, and post-match AA and C concentrations, for all 4 matches played; large to very large effect sizes (ES) are presented, together with significant differences shown as *, for *p* < 0.05. In contrast, C did not show statistically significant changes across timepoints, although large effect sizes were observed. Specifically, very large effect sizes were observed when comparing pre-match values (Match 1: 0.25 ± 0.08 µg/dL; Match 2: 0.31 ± 0.12 µg/dL; Match 3: 0.28 ± 0.08 µg/dL; Match 4: 0.21 ± 0.08 µg/dL) with both half-time (Match 1: 0.69 ± 0.26 µg/dL; Match 2: 1.13 ± 0.65 µg/dL; Match 3: 0.73 ± 0.37 µg/dL; Match 4: 0.74 ± 0.42 µg/dL) and post-match values (Match 1: 0.69 ± 0.47 µg/dL; Match 2: 0.81 ± 0.61 µg/dL; Match 3: 0.68 ± 0.26 µg/dL; Match 4: 0.71 ± 0.51 µg/dL).

Figure 1 illustrates that AA levels increased significantly from pre- to post-match in the first game (pre: 247.90 ± 232.59 U/mL; half-time: 600.62 ± 555.19 U/mL; post: 674.39 ± 693.33 U/mL; *p* < 0.001; 95% CI −0.01–1.66). A similar pattern was observed in the third match, where AA values rose significantly from pre-match (115.63 ± 124.83 U/mL) to post-match (355.61 ± 503.89 U/mL; *p* = 0.05; 95% CI −0.17–1.48).

**Figure 2:**
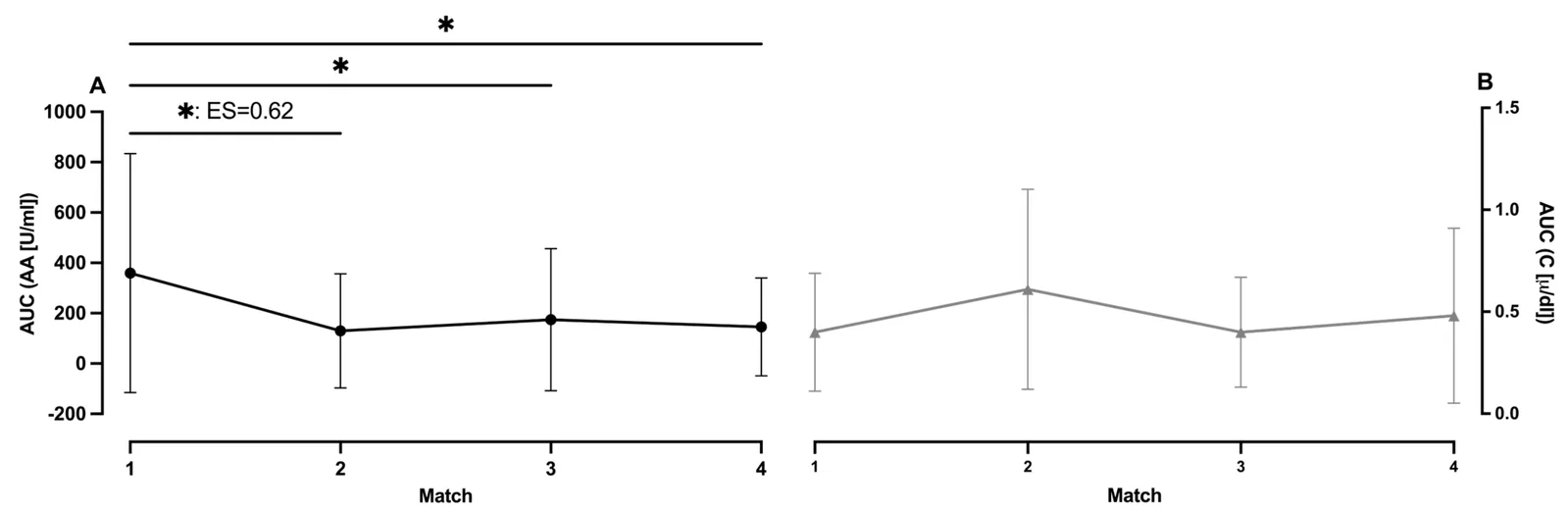
Area under the curve (AUC) for alpha-amylase (U/mL) (**A**) and cortisol (µg/dL) (**B**), calculated from pre-match, at the half-time, and post-match values showing differences between matches played (1–4). Moderate effect sizes (ES) are presented together with significant differences shown as *, for *p* < 0.05. Position 1 (black), Position 2 (light grey), Position 3 (dark grey).

To assess differences between matches, area under the curve (AUC) analyses were conducted (Figure 2). Match 1 (359.63 ± 475.33) showed significantly higher AA responses compared to the remaining matches (Match 2: 129.61 ± 226.66; Match 3: 174.33 ± 282.12; Match 4: 145.72 ± 194.84; *p* = 0.01–0.02). No significant differences were found for cortisol AUC across matches.

Position-specific analysis revealed that Position 2—forwards (0.24 ± 0.18) exhibited a smaller cortisol effect compared to Position 1—guard players (0.55 ± 0.31) and Position 3—center players (0.51 ± 0.22), whereas no positional differences were observed for AA.

**Figure 3:**
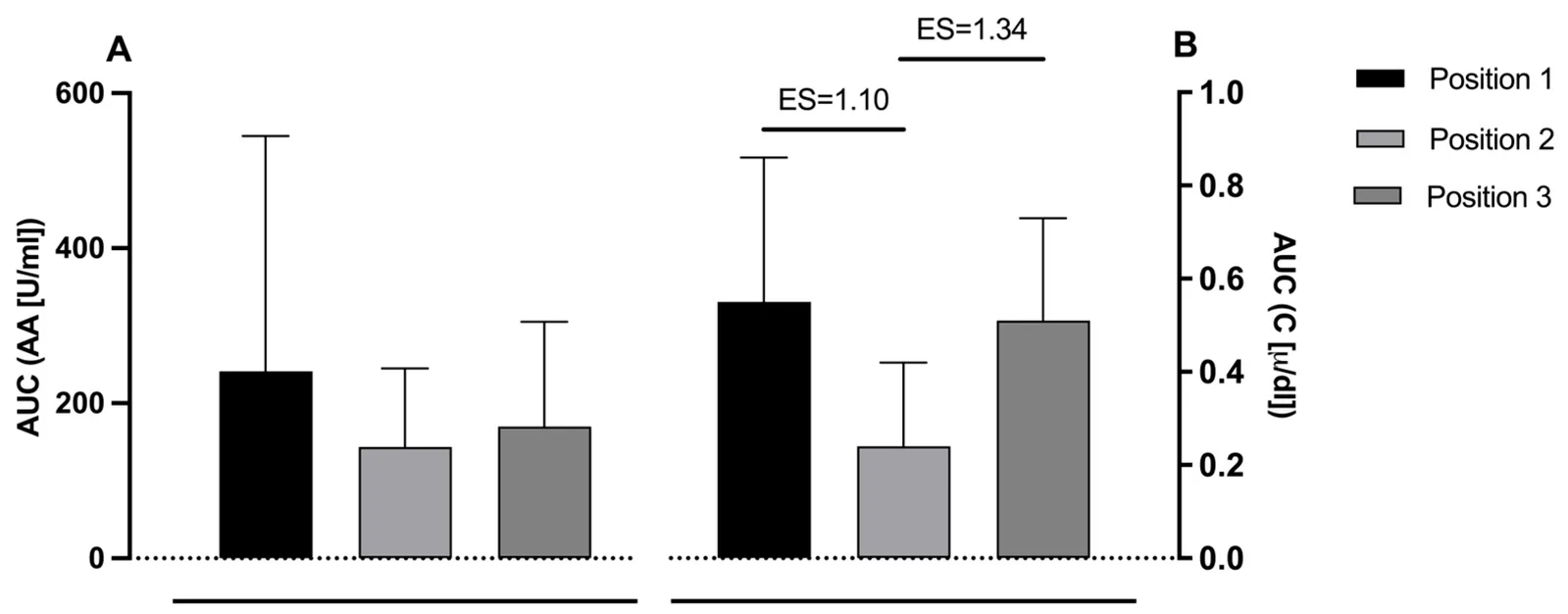
Area under the curve (AUC) for alpha-amylase (U/mL) (**A**) and cortisol (µg/dL) (**B**), used to determine differences between playing positions calculated from pre-match, at the half-time, and post-match values; large effect sizes (ES) are presented to show differences. Position 1(black)—guard players; Position 2 (light grey)—forwards; Position 3 (dark grey)—center.

**Figure 4:**
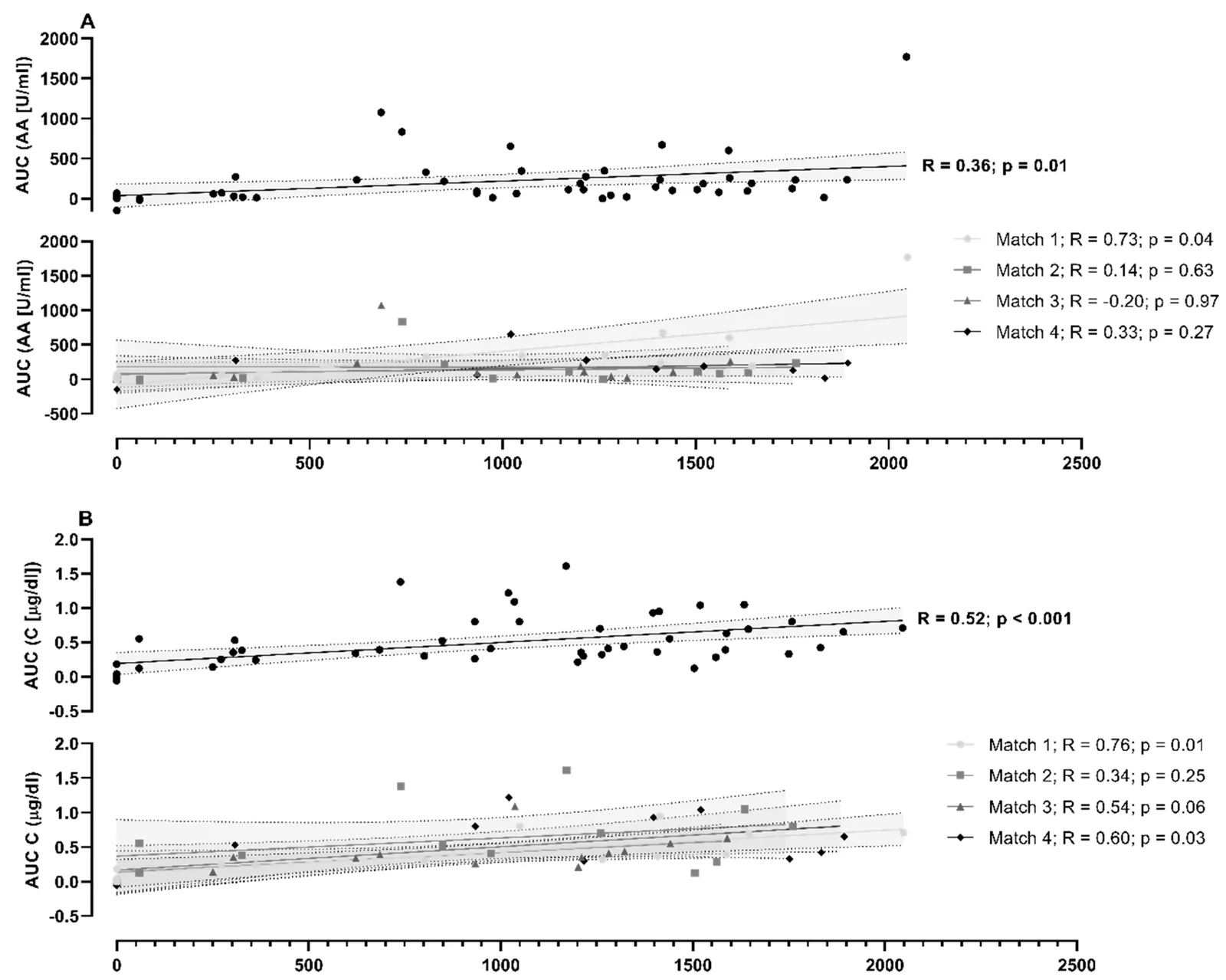
Area under the curve (AUC) for alpha-amylase (U/mL) (**A**) and cortisol (µg/dL) (**B**) in relation to playing time, with the upper panel showing the relation in all matches and the lower panels showing the relation for individual matches.

Further analyses examined the relationship between biomarkers and playing time (PT) (Figure 3). ANOVA indicated no significant differences in PT across playing positions; therefore, correlation analyses were performed on the entire sample (Figure 4). Both AA AUC and cortisol AUC were significantly correlated with PT (*p* < 0.001 and *p* = 0.01, respectively). However, when analyzed on a match-by-match basis, significant associations were limited. For AA, only the first match showed a significant correlation with PT, while for C, significant positive correlations were identified in the first and third matches.

## 4. Discussion

### 4.1. Responsiveness of Salivary Stress Biomarkers in Basketball Matches

This study suggests that playing a basketball game can significantly increase salivary biomarkers due to highly stressful conditions [20]. These results are consistent with previous literature, identifying competitive sports as a potent stressor due to the crossed influence of physical exertion, psychological pressure (e.g., competitive anxiety), and tactical demands [21,22]. Moreover, previous studies investigated changes in salivary biomarkers, including different inclusion criteria of basketball matches (e.g., final outcomes, phase of the season, comparison between simulated and official matches) [23]. Additionally, detected increases in biomarkers indicate that participation in competitive basketball elicits a measurable physiological and psychological stress response. Therefore, the findings of the present study confirm previously reported observations, also demonstrating that stress responses during basketball matches are not uniform, and the dominant source of stress may vary depending on the characteristics of each game played. A review of the literature suggests that a limited number of studies have simultaneously assessed AA and C in basketball game settings. Additionally, there is a lack of studies examining possible correlations between playing time and release of stress biomarkers, specifically AA and C. Only a limited number of studies have examined the relationship between playing time and stress biomarkers in basketball players, with available evidence largely restricted to cortisol, while comparable data for salivary alpha-amylase remain scarce [20,23]. Accordingly, this study aimed to determine changes in salivary biomarkers during basketball matches. It must be noted that because all matches and sampling sessions were conducted at approximately the same time in the late afternoon, the influence of circadian rhythm was considered consistent across all measurements and was therefore not discussed separately. Consequently, the observed post-match increases in cortisol are more likely to reflect the physiological and psychological demands of competition than diurnal variation. Study results revealed several important findings: (1) salivary biomarkers, including AA and C, respond differently depending on underlying stressors; (2) PT analysis indicated stress load in basketball matches; (3) playing position analysis showed differences in stress responses—particularly between guard and forward players, and forward and center players, with no differences between guard and center players.

### 4.2. Underlying Stressors as Modulators of Stress Pathway in Basketball Matches

The first major finding of the present study showed that stress biomarker responses may vary due to the different underlying stressors (e.g., pre-competitive anxiety and physical exertion). In some matches, higher levels of physiological stress biomarker (C) were observed without a corresponding increase in psychological stress biomarker (AA).

AA values indicated a higher increase in environments characterized predominantly by psychological stress, as reported previously [24,25]. Specifically, significantly higher AA values were observed in the first basketball match. This may be explained by the higher perceived importance of the match and pre-anxiety, respectively, as supported by elevated baseline values in basketball players prior to competition [26]. Similarly, Lim [27] noted that AA levels and perceived anxiety significantly increased preceding archery competition, with AA increasing alongside heightened anxiety during the competition. Accordingly, this suggests a possible relationship between perceived stress and elevated AA levels in sports environments. In our study, the first match was characterized by playing against a highly ranked team, since the match was crucial for securing advancement to the next stage of the competition. In this way, this match may represent a greater psychological demand, which was corroborated in correspondence with the coach.

On the other hand, C showed a tendency to respond to high-intensity physical demands in competitive environments (e.g., physical exertion) [28,29]. Given this, our finding can be explained by increased activity of the sympathetic–adrenal–medullary axis due to a higher perceived threat of failure, as confirmed by a previous study [29]. Furthermore, activation of the hypothalamic–pituitary–adrenal (HPA) axis elicits C, the primary physiological stress biomarker [30,31]. Therefore, C represents a main indicator of athletes’ physiological profile in sports settings (i.e., physiological workload) [31]. Contrary to previous studies investigating basketball matches, the analysis of the C levels in the present study showed no significant statistical differences across four matches [13,23,32]. Specifically, Moreira, McGuigan, Arruda, Freitas, and Aoki [32] reported a greater magnitude of C after official matches, when compared to simulated matches. Additionally, Kamarauskas and Conte [23] demonstrated that playing a basketball match induced increased C levels regardless of season phase (i.e., regular vs. semi-final vs. final matches), match outcome (i.e., winning vs. losing), and location (i.e., home vs. away). Additionally, a previous systematic review on testosterone and cortisol responses in basketball players indicated that C consistently reflects acute post-match stress responses [23]. Although C levels in the second match appear to be higher, this increase was not statistically significant. Moreover, such results may indicate that physical demands were most likely similar in observed matches, as can be seen in the presented values of C. Regardless, multiple studies demonstrated no significant differences in C levels across competitive matches in team sports, though some contextual factors may impact this response. Hence, the evidence supporting this finding is substantial. In addition, Casanova et al. [33] investigated elite female soccer players across four official matches and established no significant differences across all matches. Similar results were demonstrated by Moreira, Aoki, de Arruda, Machado, Elsangedy, and Okano [29] when examining professional male soccer players, and reported no significant changes in salivary C between two observed teams in a competitive training match. Moreover, a previous study analyzed male rugby athletes and established no significant changes in C levels pre- and post-competition [34].

Taken together, C showed non-statistical increase and inconsistent results across the observed matches. This might suggest that its increase may depend on contextual and situational factors. These findings indicate that performance in a basketball game is influenced by a complex interplay of multiple factors. Both psychological and physiological stress responses do not arise from a single source but are shaped by several interrelated stimuli. In our study, these responses were especially affected by game importance and by matches involving significantly greater physical contact.

### 4.3. Playing Time (PT) as an Indicator of Stress in Basketball Matches

In competitive environments, athletes are exposed to both external and internal stressors. External load refers to the cumulative demands placed on the athlete during physical activity, such as a total distance covered, frequency and intensity of physical contacts, or playing time (PT) [35]. On the other hand, internal load indicates the athlete’s individual physiological (i.e., C concentrations) and psychological (i.e., AA concentrations) responses to the abovementioned demands. Among many other factors, PT in basketball indicates the external stress experienced by players, influencing both their physical workload and stress-related responses [20]. Increased playing time in basketball likely elevates cortisol responses because prolonged exposure to high-intensity intermittent exercise increases the cumulative physiological load, exceeding the exercise intensity threshold required to stimulate cortisol secretion. This interpretation is consistent with the findings of Hill et al. (2008), who demonstrated that cortisol concentrations increase when exercise intensity and overall physiological stress surpass a critical threshold [36].

The present study demonstrated a correlation between PT and C levels. Players who played longer on the court exhibited a larger individual increase in C. In contrast to our finding, Schelling, Calleja-González, Torres-Ronda, and Terrados [20] demonstrated that basketball players with lower PT exhibited higher C, due to emotional stress induced by a smaller personal contribution to the team. As far as we are aware, more studies reporting comparable data are lacking. Therefore, the present findings from our study suggest that there is a relationship between C and physical load. Hence, longer PT increases players’ exposure to physiological stress, leading to higher C responses.

### 4.4. Differences in Stress Responses Across Playing Positions

The present study demonstrated notable differences in stress responses between playing positions. Importantly, the combined analysis of AA and C provides a more comprehensive understanding of these differences, as these biomarkers reflect distinct but complementary stress pathways—AA primarily indicating sympathetic–adrenal–medullary activation (acute psychological stress), and C reflecting hypothalamic–pituitary–adrenal axis activation (predominantly physiological load) [25,30]. In the present study, AA responses did not show clear positional differences, suggesting that acute psychological stress was relatively uniform across all playing positions. This finding is consistent with previous research in basketball and other team sports, indicating that psychological stress during competition is largely driven by situational factors such as match importance, uncertainty of outcome, and competitive pressure, rather than just positional role [28,29]. Given that all players are simultaneously exposed to the same competitive environment, it is reasonable to infer that AA, as a sensitive marker of immediate psychological arousal, reflects a shared stress response across positions.

In contrast, C responses revealed positional differences, with guards and centers exhibiting higher values compared to forward players. These findings align with the distinct functional demands associated with each position. Guards are primarily responsible for ball handling, tactical organization, and rapid decision-making under pressure, which has been associated with elevated cognitive and emotional stress [21]. This is supported byClaudio, R. et al. [28], who demonstrated that psychological states during competition are closely linked to physiological stress markers in basketball players [28]. On the other hand, centers and power forwards are exposed to frequent physical contact, rebounding, and positional play in confined spaces, which impose substantial mechanical and physiological load. Similar patterns have been observed in other team sports; for example, Crewther, B.T., et al. [34] reported elevated C responses in rugby players engaged in repeated collisions, while Moreira, A. et al. [32] and Cabarkapa, D. et al. [35] highlighted the relationship between external load and internal physiological responses in basketball. Interestingly, the absence of significant differences between guards and centers suggests that different types of stressors—primarily psychological in guards and physical in centers—may elicit comparable overall C responses [32,35,37]. This observation is supported by previous literature demonstrating that both psychological and physiological demands can activate similar endocrine pathways and produce comparable hormonal outputs [25,30]. Additionally, similar findings have been reported in soccer, where C responses tend to vary with physical workload, while psychological stress remains relatively consistent across positions [33]. The lower C response observed in forward players may be explained by their more balanced role, involving moderate levels of both cognitive and physical demands. This agrees with previous findings suggesting that players with hybrid or less specialized roles experience lower overall stress compared to positions characterized by extreme physical or cognitive loads [32,35]. Overall, the present findings are consistent with existing literature across basketball and other team sports, emphasizing that stress responses are both position-dependent and specific to stress response pathways. While AA reflects a shared, context-driven psychological stress response across all players, C appears to be more sensitive to position-specific demands, integrating both cognitive and physical stressors. These results highlight the importance of a multidimensional approach to stress monitoring, as the use of both biomarkers allows for a more precise interpretation of the underlying sources of stress in relation to playing position.

## 5. Conclusions

The present study demonstrates the responsiveness of stress-related biomarkers—AA and C—in basketball players across four matches. The observed variability in AA and C concentrations suggests that stress responses are not uniform, but rather influenced by a complex interaction of physiological and psychological stimuli inherent to competitive basketball. Specifically, contextual and situational factors were shown to play a significant role. Playing time emerged as a key determinant of physiological stress, with greater playing time associated with higher cortisol levels, indicating increased cumulative workload. In contrast, playing position revealed no significant differences in AA, whereas C levels varied by position, with guards and centers exhibiting higher values than forwards. This finding may reflect position-specific demands, with guards potentially experiencing greater cognitive demands and centers greater physical contact; however, these interpretations should be considered speculative and warrant confirmation in future studies. 

A limitation of the present study is the relatively small sample size (*n* = 12), which may have reduced the statistical power and limited the generalizability of the findings to broader populations of athletes. Although cortisol exhibited large effect sizes, these differences did not reach statistical significance, likely due to the limited sample size and the resulting low statistical power. Therefore, the observed findings should be interpreted as potentially meaningful from a practical perspective but require confirmation in studies with larger samples. A limitation of this study is its observational design, which precludes causal inferences and allows only the identification of associations between the investigated variables. Another limitation of the present study is that psychological stress was not assessed using validated psychological instruments (e.g., anxiety questionnaires), and therefore the interpretation of stress responses relied primarily on indirect physiological markers and coach observations. An additional limitation of this study is the small number of centers included in the sample (n = 2), which may have reduced the reliability of positional comparisons and limited the generalizability of findings related to playing position. An additional limitation of this study is that the relatively small number of observations within individual matches may have reduced the statistical power to detect consistent associations between playing time and stress biomarkers. 

Overall, these findings highlight the value of simultaneously monitoring AA and C to obtain a broader understanding of stress responses in basketball. From a practical perspective, this approach offers valuable insights for coaches and sports practitioners for potential application, which might lead to more precise monitoring of individual responses to both psychological and physiological demands. Such information may support improved and specifically tailored training load management, optimized recovery strategies, and a reduced risk of overtraining and injury. This potential application requires more analyses and further confirmation in order to be proposed as a reliable recommendation.

## Data Availability

The original contributions presented in this study are included in the article. Further inquiries can be directed to the corresponding author.
